# Of ‘Disgrace’ and ‘Pain’ – Corticolimbic Interaction Patterns for Disorder-Relevant and Emotional Words in Social Phobia

**DOI:** 10.1371/journal.pone.0109949

**Published:** 2014-11-14

**Authors:** Inga Laeger, Christian Dobel, Britta Radenz, Harald Kugel, Kati Keuper, Annuschka Eden, Volker Arolt, Pienie Zwitserlood, Udo Dannlowski, Peter Zwanzger

**Affiliations:** 1 Department of Psychiatry, University of Münster, Münster, Germany; 2 Institute for Biomagnetism and Biosignalanalysis, University of Münster, Münster, Germany; 3 Department of Clinical Radiology, University of Münster, Münster, Germany; 4 Institute for Psychology, University of Münster, Münster, Germany; 5 Department of Psychiatry, University of Marburg, Marburg, Germany; 6 kbo-Inn-Salzach-Hospital, Wasserburg am Inn, Germany; West China Hospital of Sichuan University, China

## Abstract

Limbic hyperactivation and an impaired functional interplay between the amygdala and the prefrontal cortex are discussed to go along with, or even cause, pathological anxiety. Within the multi-faceted group of anxiety disorders, the highly prevalent social phobia (SP) is characterized by excessive fear of being negatively evaluated. Although there is widespread evidence for amygdala hypersensitivity to emotional faces in SP, verbal material has rarely been used in imaging studies, in particular with an eye on disorder-specificity. Using functional magnetic resonance imaging (fMRI) and a block design consisting of (1) overall negative, (2) social-phobia related, (3) positive, and (4) neutral words, we studied 25 female patients with social phobia and 25 healthy female control subjects (HC). Results demonstrated amygdala hyperactivation to disorder-relevant but not to generally negative words in SP patients, with a positive correlation to symptom severity. A functional connectivity analysis revealed a weaker coupling between the amygdala and the left middle frontal gyrus in patients. Symptom severity was negatively related to connectivity strength between the amygdala and the ventromedial prefrontal and orbitofrontal cortex (Brodmann Area 10 and 11). The findings clearly support the view of a hypersensitive threat-detection system, combined with disorder-related alterations in amygdala-prefrontal cortex connectivity in pathological anxiety.

## Introduction

Social phobia (SP), also referred to as social anxiety disorder, is characterized by an exaggerated fear of being negatively evaluated by others in social or performance situations (DSM IV-TR, [1]). It is one of the most prevalent and burdening anxiety disorders [2]–[4]. Within the last years, neuroimaging studies have provided valuable information regarding the neural substrates of altered emotion processing in SP, with a focus on the limbic system (e.g. the amygdala as the most prominent structure), and on executive regulative brain systems such as the prefrontal cortex. Theories assume that the functional balance between these neural systems may be impaired in pathological anxiety, possibly leading to a less efficient top-down control of the ‘emotional’ amygdala [5]–[7].

Regarding the limbic system, amygdala hyperactivation to disorder-relevant stimuli is a well-established finding in SP. The socially relevant stimuli are frequently operationalized as faces with aversive expressions [8]–[16], for review see [7]. Studies reporting correlations between amygdala activation and symptom severity underlined the crucial role of this brain region [12], [17]–[19].

Although words play an important role in research on biased information processing in SP (e.g., modified emotional-Stroop tasks [20], [21]), and have several methodological advantages, only a few fMRI-studies so far have used verbal material as emotional stimuli. Schmidt and colleagues recently showed increased amygdala activation in SP for disorder-specific words [22], yet without comparing these stimuli to other emotional and especially generally negative words. Similarly Blair and co-authors reported increased amygdala activation to comments related to the patients themselves, or to other persons [23], and to descriptions of social transgressions [24]. However, alterations of amygdala activation have been found in many other anxiety disorders and symptoms, such as specific phobia [25], [26], generalized anxiety disorder [27], and increased trait anxiety [28]–[30]. Thus, this neural correlate is not specific for SP, and does not help clarifying the mechanisms underlying this disorder *per se*.

In comparison to such limbic changes, less is known about alterations on a cortical level and again, only little in response to verbal stimuli. Some studies reported decreased frontal cortex activation in SP [15], [31], [32], which is compatible with the idea of a failure of prefrontal regions in down-regulating the amygdala. Accordingly, using resting-state or task-related functional connectivity (FC) analyses, several authors reported a decrease in functional coupling between the amygdala and emotion-regulation-related frontal brain in social phobia [33]–[35]. For example, Goldin and colleagues studied corticolimbic activation and coupling in response to individual autobiographical negative self-beliefs, while patients and controls were instructed to either perceive or down-regulate their feelings. For the reappraisal condition they found a later onset of activity in emotion-regulation related prefrontal brain structures, and fewer prefrontal regions inversely coupled with the left amygdala in social phobia patients [33]. Other findings indicated that not only corticolimbic but also connectivity within the prefrontal cortex is reduced in SP [36]. On the other hand, there are also contradicting results indicating increased frontal activation in SP [22]–[24], [37]–[39]. For example, Brühl et al. studied brain activation during the anticipation of generally emotional pictures and found increased medial prefrontal cortex and dorsolateral prefrontal cortex, but decreased orbitofrontal cortex (OFC) activation during the anticipation of negative versus neutral images in social phobia [18].

From a methodological point of view, the frequently used facial stimuli have one important disadvantage: although it seems most probable that SP subjects react alarmed to angry or contemptuous faces due to their anxiety of being criticized, these faces are negative stimuli in general, and disorder-specific biases cannot be determined. This constitutes a problem, since increased amygdala activation in SP occurs even for neutral faces [13] and for generally negative stimuli [18], [19]. Thus, it would be highly advantageous to use not only disorder-relevant, but disorder-specific material, and to compare this with generally negative stimuli, to understand better the neural mechanisms underlying the specific disorder. Verbal stimuli provide this opportunity, while they have never been studied in detail using fMRI in social phobia patients. Furthermore, up to now no study has analyzed the association between disorder severity and corticolimbic coupling while patients are confronted with emotional and disorder-specific words.

Thus, the current study aimed to answer two questions. First, do SP patients, as compared to HC, exhibit increased amygdala activation, and a positive association with disorder severity, only to disorder-specific words or also to generally negative emotional stimuli, as was shown by [18], [19]? Secondly, do differences exist between patients and controls in the functional coupling between amygdala and frontal cortex during word reading and, more importantly, does the patients' disorder severity correlate with this connectivity?

## Materials and Methods

### Participants

Twenty-five patients with social phobia and 25 healthy controls took part in this study.

Due to the higher prevalence of social phobia in women [40] and given that there are marked differences between men and women in neural response patterns to emotional words [41], only female participants were included in the current sample. All participants responded to local newspaper ads and were screened by an experienced clinical psychologist. All included patients fulfilled the criteria of a current social phobia according to DSM-IV [1], as confirmed with the SCID interview [42]. None had a comorbid diagnosis of a current phase of a major depression or a generalized anxiety disorder, nor a life-time diagnosis of psychotic symptoms and substance abuse. Comorbid diagnoses in the patients were currently remitted depressive disorder (*n* = 3), currently remitted single major depressive episode (*n* = 2), currently remitted anorexia nervosa (*n* = 1), and specific phobia (*n* = 3). Only one patient received psychotropic medication (15 mg Citalopram every second day, excluding this patient would not alter the pattern of results) and *n* = 10 patients had former or current psychotherapeutic treatment such as cognitive behavioral therapy (*n* = 7) or other forms (*n* = 3). Healthy participants had no life-time history of any psychiatric disorder or psychotropic medication. Exclusion criteria for all participants were neurological illnesses or a history of seizures or head trauma, intake of benzodiazepines, head movements of >2 mm and/or 2° and the general MRI-contraindications. All participants were German native speakers, and had normal or corrected-to-normal vision. Except for 6 persons in each group, all participants were right-handed (according to a slightly adapted version of the handedness questionnaire [43]).

Before scanning, all subjects filled in the German versions of the Beck-Depression Inventory, BDI [44], the Trait- and State-version of the State-Trait Anxiety Inventory, STAI [45] and the Social Phobia Scale (SPS) and Social Interaction Anxiety Scale (SIAS). The SPS assesses the fear of being scrutinized by others during several activities (e.g., drinking, speaking) while the SIAS measures fears of interacting with others [46]. See Table 1 for an overview on the above and other sociodemographic and questionnaire data of the two groups.

**Table 1:** Mean differences for patients with social phobia (SP) and healthy controls (HC) concerning age, verbal intelligence, depression (BDI), Trait- and State anxiety (STAI-T and STAI-S) and social phobia symptoms (SPS/SIAS).

	SP *M*± SD	HC *M*± SD	*t*-value	*p*-value (2-tailed)
AgeEducation yearsVerbal intelligence^a^BDISTAI-TSTAI-SSPSSIAS	29.76±8.2614.68±1.82111.32±13.1911.92±6.8653.12±9.3541.16±7.9237.16±16.2445.56±14.52	29.36±9.8214.96±1.59111.44±12.451.44±1.6430.56±4.4330.56±4.132.64±2.788.40±6.69	−0.160.580.03−7.43−10.91−5.93−10.47−11.62	0.880.570.97<0.001**<0.001**<0.001**<0.001**<0.001**

*M* =  Mean; SD  =  standard deviation. BDI, Beck Depression Inventory; STAI-T, State-Trait Anxiety Inventory, Trait version; STAI-S, State-Trait Anxiety Inventory, State version; SPS, Social Phobia Scale; SIAS, Social Interaction Anxiety Scale.

a Assessed with the Mehrfachwahl-Wortschatz-Intelligenztest [47].

### Ethics statement

All procedures were approved by the Ethics Committee of the Medical Faculty of the University of Münster. The ethical standards of the Declaration of Helsinki were met. All participants provided written informed consent and received financial compensation for their participation. Patients were additionally offered psychological consultation.

### Task and procedures

A total of 96 German nouns, 24 negative (e.g., pain, victim), social-phobia related (e.g., disgrace, audience), positive (e.g. holidays, baby), and neutral ((e.g. pencil, arm), all examples translated from actually used German words) was used in this study. Negative, positive, and neutral words constituted a subset of the stimuli from Kissler and colleagues [48], which have already been shown to induce amygdala activation in healthy subjects [30]. Social-phobia related nouns were taken from Schmidt et al. [22], who demonstrated appropriate limbic and frontal activations in response to these stimuli in 19 SP subjects. All word categories were matched for word length (letters: *M*_neg_ = 7.88, *M*_SP-rel,_ = 9.25, *M*_pos_ = 7.75, *M*_neu_ = 8.17; all t<1.69, all p>0.05) and frequency of use in written German according to the CELEX database, [49] (*M*_neg_ = 28.88, *M*_SP-rel._ = 50.92, *M*_pos_ = 55.25, *M*_neu_ = 52.75; all t<1.46, all p>0.05). The words were furthermore rated by all participants for valence, arousal and anxiety induction using a nine-point-Likert scale (SAM, Self-Assessment Manikin, [50]), ranging from 1 =  not pleasant/arousing/anxiety-inducing to 9 =  very pleasant/arousing/anxiety-inducing.

The fMRI-paradigm was programmed with Presentation Software (Version 12.1, Neurobehavioral Systems, Inc., Albany, CA, USA; www.neurobs.com). Words were presented in white colour in the centre of a black screen in alternating 15 s blocks of 12 words per block. Presentation time was 1000 ms per word, with a fixed interstimulus interval of 250 ms. The blocks of words were presented in a pseudo-randomized order. A 10 s resting phase (white fixation cross in the centre of a black screen) was following each block of words. In all, the paradigm took 400 s (6:40 min), and consisted of 8 word blocks (2 negative, 2 SP-related, 2 positive, 2 neutral). The stimuli were projected onto a screen at the rear end of the MR tunnel, using a projector shielded against RF interference. Each block was presented two times and the participants were instructed to read the words attentively. No further instruction was given.

### Image acquisition

A 3 Tesla scanner (Gyroscan Intera T3.0, Philips Medical Systems, Best, NL) equipped with Quasar Dual gradients (maximal gradient strength 40 mT/m, maximal slew rate 200 mT/m/ms) was used to acquire MRI data. For spin excitation and resonance signal acquisition, a circularly polarized transmit/receive birdcage head coil with an HF reflecting screen at the cranial end was used. T2* functional data were acquired using a single-shot echo planar imaging (EPI) sequence (whole brain coverage, TE = 30 ms, TR = 2.5 s, FA = 90°, 40 slices, slice thickness 3.6 mm, no gap, matrix 64×64, FOV 230 mm, in-plane resolution 3.6 mm×3.6 mm). The slices were tilted 25° from the anterior commissure/posterior commissure line to minimize drop out artefacts in the orbitofrontal and mediotemporal regions.

### Data analysis

*Behavioral rating data* for valence-, arousal-, and anxiety induction were analysed using 2×4 repeated-measures analyses of variance (ANOVA), with group (patients versus controls) as between-subject factor and word category (negative, SP-related, positive, neutral) as within-subject factor within the general linear model (version 20, SPSS Inc., USA). The polarity of valence ratings was reversed for the ANOVA analysis, resulting in a range from 1 =  very pleasant to 9 =  very unpleasant.

*Functional-imaging data* were analysed using Statistical parametric mapping (SPM8, http://www.fil.ion.ucl.ac.uk/spm). Images were realigned and unwarped, spatially normalised to standard MNI space (Montreal Neurological Institute), and smoothed with a Gaussian kernel of 6 mm full width at half maximum (FWHM). Onsets and durations of the different word conditions were modelled with a canonical hemodynamic response function based on the general linear model. For all subjects individual fixed-effects first-level contrasts including the conditions negative > neutral, SP-related > neutral, and positive > neutral were calculated, and the resulting contrast images were entered in the 2^nd^ level (group) random-effects analysis.

According to our main hypotheses, a region of interest (ROI) analyses of the bilateral amygdala (defined according to the AAL Atlas [51]) dilated by 1 mm in radius in order to avoid missing relevant structures using the WFU pickatlas [52]) was performed in addition to a whole-brain analysis to compare activations for each word category as compared to the neutral words baseline. For this purpose, the corresponding individual contrast maps were entered into a (3×2) analysis of variance (ANCOVA), using the full factorial model implemented in SPM8, with word category as the within-subject factor and group as between-subject factor. Given that anxiety induction ratings differed between patients and controls for the neutral word category, which served as a baseline condition for the analysis, each participant's mean rating value was entered as a covariate of no interest in the model, to ensure that functional differences between patients and controls were not due to group differences with respect to the perceived anxiety induction by neutral words. The model was used to calculate the main effects of group, word category, and the crucial group x word category interaction. Following, according to our hypothesis, a planned between-group comparison of amygdala activation for the contrast of SP-related (> neutral) versus negative (> neutral) words was conducted in addition to explorative post-hoc t-tests for other within-group (effects of word condition) and between-group comparisons.

A regression analysis was conducted for the hypothesis on the relation between social phobia severity and amygdala activation. To study the influence of the stimuli's disorder-specificity, we correlated both amygdala activation for the SP-related > negative and negative > neutral word condition, respectively, with each patient's SPS- and SIAS-score using a voxel-wise region of interest (ROI) approach.

Moreover, a functional connectivity (FC) analysis was conducted, reflecting the covariation of activity in a defined seed region with one or more other brain areas during the time course of the experiment [53]. Based on our *a priori* hypothesis about corticolimbic interaction, volumes of interest (VOI) were extracted from the left and right amygdala separately as seed region in this analysis. For both, left and right amygdala seed separately, fixed-effects first-level models were conducted including the experimental conditions as nuisance regressors. The resulting contrast images of positive functional connectivity were entered in two-sample t-tests to compare the strength of functional connectivity between SP and HC. Furthermore, the patients' individual contrast maps were correlated with SPS- and SIAS-scores. A mask of the whole frontal lobe (defined according to the WFU PickAtlas [52]) including all prefrontal areas, was used for the FC analysis. Again, an additional whole-brain analysis was conducted for the group comparison of functional connectivity to ascertain that our analysis would not miss relevant structures outside the frontal lobe ROI.

In order to control for multiple statistical testing, all group results were calculated with a combined height and extend threshold based on Monte-Carlo simulations, as implemented in the AlphaSim procedure [54]. A corrected false-positive detection rate for the amygdala region of interest analysis at *p*<0.05 was maintained, with a cluster extent (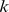) empirically determined by computing 1000 simulations (yielding 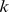 = 46 for the bilateral amygdala). The functional connectivity analysis was conducted at *p*<0.001, using a mask of the whole frontal lobe [52] (yielding 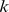 = 29 voxels as the empirically determined cluster extent). For the additional whole-brain group comparison of task-related activation and functional connectivity, a threshold of *p*<0.001 was chosen, resulting in an empirically determined cluster extent of 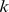 = 37 voxels. For post-hoc t-tests subsequent to significant main or interaction effects in the group x word category ANCOVA, an uncorrected threshold of *p*<0.005 was selected, representing a Bonferroni-corrected 0.05 probability.

## Results

### Rating data for verbal stimuli

The analyses of rating data for negative, SP-related, positive, and neutral words revealed significant main effects for the between-subject factor group (valence: *F*_1,48_ = 21.33, *p*<0.001, *η^2^*_p_ = 0.31; anxiety induction: *F*_1,48_ = 24.76, *p*<0.001, *η^2^*_p_ = 0.34). SP patients showed more negative valence (*M_SP_* = 5.27 vs. *M_HC_* = 4.76) and higher anxiety induction ratings (*M_SP_* = 3.25 vs. *M_HC_* = 2.09) than controls. Significant main effects were also found for the within-subject factor word category (valence: *F_2.36, 113.18_* = 681.63, *p*<0.001, *η^2^*_p_ = 0.93, arousal: *F_2.52, 120.76_* = 141.25, *p*<0.001, *η^2^*_p_ = 0.75, anxiety induction: *F_2.04, 98.04_* = 116.36, *p*<0.001, *η^2^*_p_ = 0.71). Post-hoc analyses revealed that all word categories differed significantly from each other in their valence (all *t*>5.99, all *p*<0.001) and arousal (all *t*>3.38, all *p*<0.05), except for arousal ratings for negative vs. SP-related, and negative vs. positive words (all *t*<2.31, all *p*>0.15). There were also significant differences between all word categories regarding their anxiety induction (all *t*>3.49, all *p*<0.05), except for negative versus SP-related words (*t* = 2.45, *p* = 0.11). Most importantly, there were significant group x word category interactions for all three ratings (valence: *F_2.36, 113.18_* = 15.06, *p*<0.001, *η^2^*_p_ = 0.24, arousal: *F_2.52, 120.76_* = 11.97, *p*<0.001, *η^2^*_p_ = 0.20, anxiety induction: *F_2.04, 98.04_* = 26.20, *p*<0.001, *η^2^*_p_ = 0.35). Post-hoc *t*-tests revealed that compared to HC, patients rated SP-related words as more negative, more arousing and more anxiety-inducing. Additionally, patients rated neutral and positive words as more anxiety-inducing than HC (see Table 2 for details). Correlational analyses between clinical measures and rating data for negative, SP-related, positive, and neutral words in the patients group can be found in Table 3.

**Table 2:** Results of post-scanning ratings of SP-related, negative, positive, and neutral words for valence, arousal, and anxiety induction by social phobia patients (SP) and healthy controls (HC).

	SP *M*± SD	HC *M*± SD	*t*-value	*p*-value (2-tailed)
*Valence*				
SP-relatedNegativePositiveNeutral	6.10±0.887.78±0.452.42±0.754.78±0.59	4.60±0.657.62±0.632.13±0.734.68±0.44	6.871.011.420.64	<0.001**0.320.160.53
*Arousal*				
SP-relatedNegativePositiveNeutral	6.04±1.505.55±1.155.69±1.781.99±0.67	4.03±1.494.92±1.715.90±1.741.98±0.99	4.751.530.420.08	<0.001**0.130.680.93
*Anxiety induction*				
SP-relatedNegativePositiveNeutral	5.06±1.614.45±1.491.92±0.861.57±0.47	2.01±1.023.71±1.701.38±0.471.24±0.38	7.981.642.732.69	<0.001**0.11<0.05*<0.05*

*M* =  Mean; SD  =  standard deviation; higher scores indicate word ratings as more negative, arousing, and anxiety-inducing.

**Table 3:** Correlations between the clinical measures and the behavioral rating data for negative, SP-related, positive, and neutral words within the group of social phobia patients (Pearson's correlation/p-value (2-tailed)).

	SPS	SIAS	BDI	STAI-T	STAI-S
*Valence*					
SP-relatedNegativePositiveNeutral	**0.41/0.04**0.28/0.170.07/0.730.33/0.11	**0.67/<0.001**0.16/0.440.25/0.230.14/0.51	0.32/0.120.27/0.190.03/0.89−0.11/0.62	**0.53/0.006**0.18/0.380.30/0.14−0.02/0.93	**0.55/0.005**0.19/0.37**0.45/0.02**0.38/0.06
*Arousal*					
SP-relatedNegativePositiveNeutral	**0.43/0.03**0.25/0.24−0.04/0.870.13/0.53	**0.69/<0.001**0.23/0.27−0.01/0.96**0.41/0.04**	0.39/0.060.34/0.090.08/0.72**0.44/0.03**	**0.49/0.01**0.33/0.110.12/0.57**0.43/0.03**	**0.52/0.008** **0.42/0.04**0.08/0.69**0.56/0.003**
*Anxiety induction*					
SP-relatedNegativePositiveNeutral	0.38/0.060.11/0.610.08/0.690.23/0.26	**0.65/<0.001**0.06/0.770.28/0.180.39/0.06	**0.48/0.01** **0.43/0.03** **0.40/0.04**0.32/0.12	**0.45/0.02**0.28/0.17**0.48/0.02** **0.41/0.04**	0.39/0.050.16/0.44**0.40/0.04** **0.49/0.01**

SPS, Social Phobia Scale; SIAS, Social Interaction Anxiety Scale; BDI, Beck Depression Inventory; STAI-T, State-Trait Anxiety Inventory, Trait version; STAI-S, State-Trait Anxiety Inventory, State version. Significant correlations are displayed in bold letters.

### Region of interest (ROI) analysis of the amygdala

**Figure 1:**
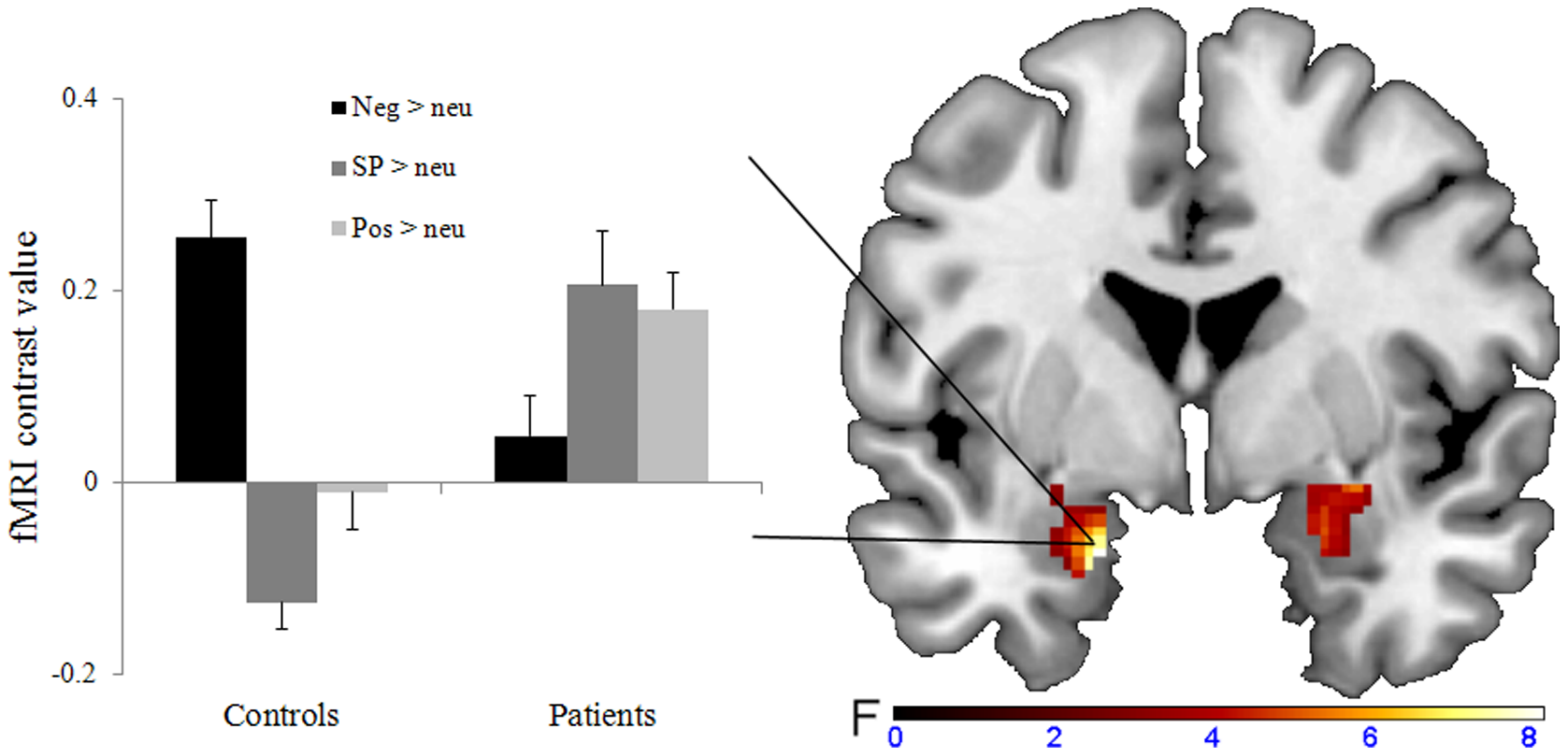
Region of interest analysis of the amygdala. Left: bar graphs depicting the mean contrast value for negative, SP-related, and positive versus neutral words extracted from x = −18, y = 0, z = −24. Error bars, SEM. Right: coronal view (y = 0), depicting the significant group x word category interaction in the bilateral amygdala.

The ANCOVA yielded no significant main effect of word category or group within the amygdala. However, there was an interaction between word category and group within the left (x = −18, y = 0, z = −24, *F*_2, 143_ = 8.16, *p*<0.001 corrected, *k* = 111 voxels) and right amygdala (x = 28, y = −4, z = −12, *F*_2, 143_ = 6.23, *p*<0.001 corrected, *k* = 155 voxels) (Figure 1). The planned comparison of the main conditions of interest revealed that patients as compared to controls showed a stronger activation of the left and right amygdala in response to SP-related (> neutral) versus negative (> neutral) words (left amygdala: x = −18, y = −2, z = −20, *t*_143_ = 3.72, *p*<0.001 Bonferroni-corrected, 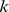 = 61 voxels; right amygdala: x = 28, y = −6, z = −14, *t*_143_ = 3.43, *p*<0.001 Bonferroni-corrected, 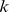 = 106 voxels). There were no significant clusters for this contrast when comparing controls to patients. Please see Table 4 for results of the additional between- and within-group post-hoc comparisons within the amygdala and Table S1 for whole-brain results of the ANCOVA.

**Table 4:** Significant results for post-hoc comparisons of the significant word-category x group interaction effect in the amygdala, conducted at p = 0.005 uncorrected, representing a Bonferroni-corrected 0.05 probability.

Amygdala activation	Side	Cluster Size	x	y	z	*T*_143_	*p*-value
*Within-group comparisons*							
HC: neg_> neu_ *vs.* pos_> neu_ words	L	9	−20	2	−24	3.15	0.001
HC: neg_> neu_ *vs.* SP-rel._> neu_ words	L	88	−18	−2	−20	3.76	<0.001
	R	17	28	−8	−14	3.24	0.001
	R	1	24	−2	−22	2.65	0.005
HC: pos_> neu_ *vs.* SP-rel._> neu_ words	L	2	−22	−8	−16	2.63	0.005
SP: SP-rel._> neu_ *vs.* neg_> neu_ words	R	2	36	6	−20	3.24	0.001
	R	3	26	8	−18	2.94	0.002
*Between-group comparisons*							
SP *vs.* HC: pos_> neu_ words	L	5	−22	2	−28	3.09	0.001
SP *vs.* HC: SP-rel._> neu_ words	R	50	24	−2	−14	3.36	0.001
	L	71	−26	−6	−12	3.13	0.001

Coordinates are given in MNI space.

SP, Social Phobia patients; HC, healthy controls.

**Figure 2:**
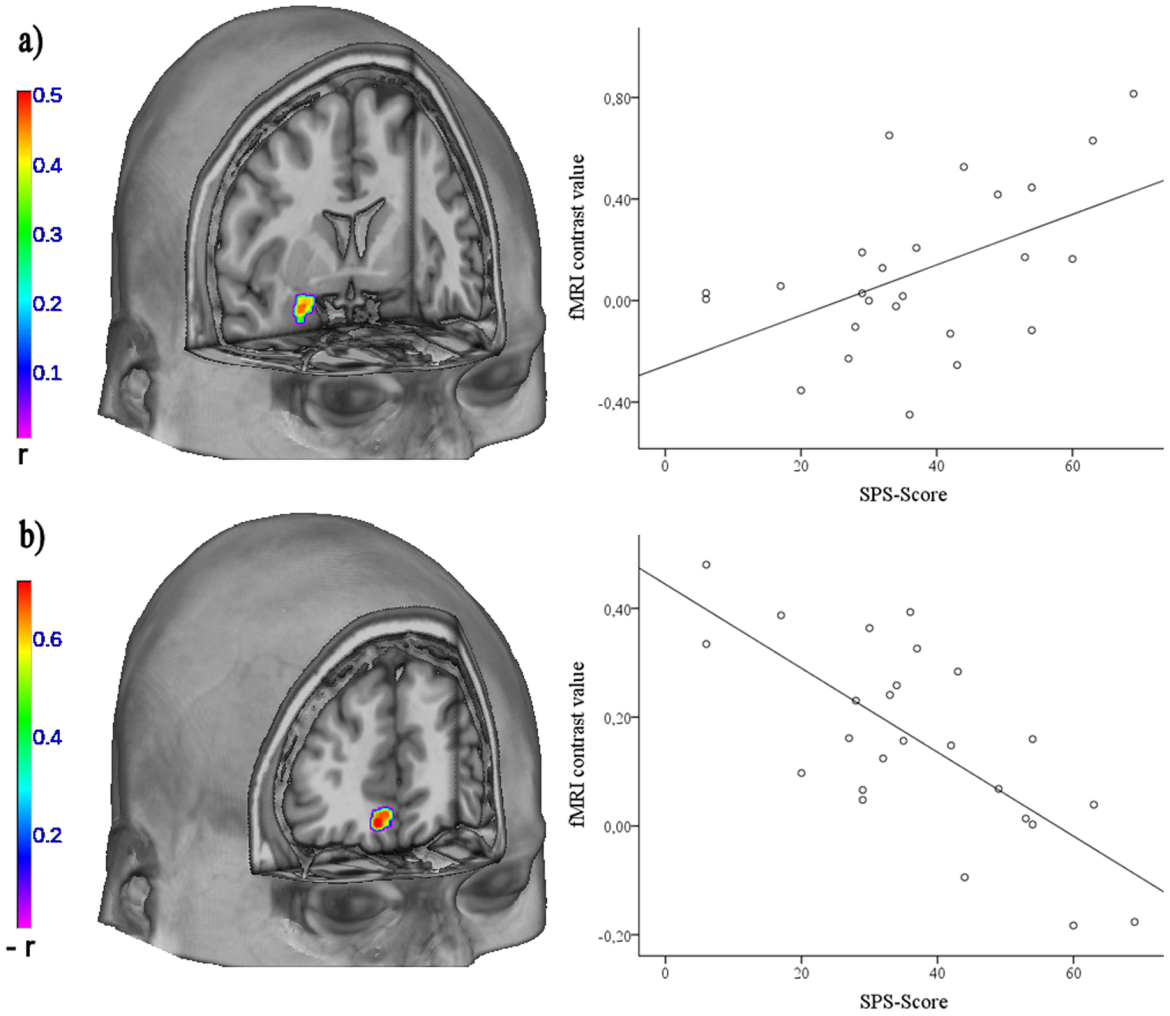
Correlations with disorder severity. **a**) Left: Association of the patients' SPS-scores with right amygdala responsiveness to SP-related versus negative words, rendered on an anatomical template in MNI-space. Color bar, correlation coefficient *r*. Right: Scatter plot depicting the positive correlation of the mean cluster activation values (left panel) and the SPS-scores. **b**) Left: Association of the patients' SPS-scores and the functional connectivity between the left amygdala and the right BA 10 and 11, rendered on an anatomical template in MNI-space. Color bar, correlation coefficient −*r*. Right: Scatter plot depicting the negative correlation of the mean cluster activation values (left panel) and the SPS-scores.

The regression analysis revealed that for the contrast SP-related versus negative words, activation of the right amygdala was positively associated with the patients' SPS-scores (x = 24, y = 6, z = −16, *t*_23_ = 2.80, *p* = 0.038 corrected, 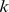 = 49 voxels, *r* = 0.50; Figure 2a). There was no correlation between SIAS-scores and amygdala activation. Correlating the patients' amygdala activation for the contrast of negative versus neutral words with the SPS and SIAS revealed no significant results.

### Functional connectivity analysis

Healthy controls demonstrated a stronger functional coupling between the right amygdala and the left middle frontal gyrus (x = −36, y = 28, z = 52, *t*_48_ = 4.30, *p* = 0.018 corrected, 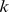 = 35 voxels, Brodmann Area (BA) 8). There were no group differences in amygdala-frontal coupling when using the left amygdala as seed region. The additional whole-brain analysis revealed no other significant group differences for the left or right amygdala seed.

Disorder severity of social phobia patients was negatively associated with functional coupling between the left amygdala and the orbital part of the medial frontal gyrus for the SPS (x = 8, y = 42, z = −12, *t*_23_ = 4.92, *p* = 0.006 corrected, 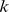 = 42 voxels, *r* = −0.72, BA 10, 11, see Figure 2b). For the SIAS, there was a negative correlation between symptom severity and the coupling between the left amygdala and the superior and middle frontal gyrus (x = −24, y = 50, z = 14, *t*_23_ = 6.41, *p* = <0.001 corrected, 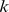 = 146 voxels, *r* = −0.80, BA 10).

## Discussion

The first aim of the current study was to replicate the observed limbic correlates of an increased sensitivity in social phobia for possibly threatening stimuli with overall negative and social-phobia related verbal stimulus material. Secondly, with a functional connectivity analysis, we wanted to provide additional evidence regarding the impaired functional relation between crucial emotion-processing subcortical regions (amygdala) and prefrontal brain areas. A detailed regression analysis with the patients' symptom severity scores was carried out in order to better understand the relation between illness severity and brain processes.

Patients with social phobia showed increased amygdala activation for SP-related (> neutral) versus overall negative (> neutral) words. While activation to SP-related words was stronger in patients than in HC, there were no group differences regarding the activation to generally negative words. This effect remained stable when group differences in anxiety-induction ratings for the neutral word baseline were taken into account. Additionally, there was a positive association between illness severity and amygdala activation in response to SP-related as compared to negative words, but not to negative versus neutral words.

The finding of a hypersensitive amygdala in patients and a positive association between amygdala activation and disorder severity concur with several other studies [8], [9], [12], [14], [17], [19], [23], [32]. In accordance with the results from Schmidt and colleagues [22], our data underline the appropriateness of linguistic stimuli in clinical research. Schmidt and colleagues also found amygdala hyperactivation in the patient group for the disorder-relevant as compared to neutral words. This only held for an indirect task of deciding whether the presented word was a noun or not. Our findings extend these results, by showing that their disorder-relevant nouns also caused amygdala hyperactivation in a passive viewing task. More important, the ROI and the correlation analysis showed that amygdala activation in social phobia is altered for disorder-specific words only, but not for negative emotional stimuli in general (as reported by [18], [19]). However, it cannot be ruled out that this finding resulted from the actual use of both disorder-relevant and generally negative stimuli, contrary to the paradigms of [18], [19]. This might have led to a decreased salience of the other word categories, which might also account for the lack of significant differences between negative and positive words in the patient group. These differences were however found in HC and in the rating data, underlining the generally good discrimination between the word categories. Moreover, patients with social phobia exhibited a stronger activation than HC of the left amygdala in response to positive (> neutral) words. Although the processing of positive stimuli in social phobia was not the focus of our study, this result is of certain interest. It is in accordance with findings by Straube and colleagues [11], who reported increased amygdala activation in SP to happy facial expressions. Straube et al. concluded that the amygdala might also be associated with the processing of safety signals in social phobia under less demanding task conditions [11], which fits with the passive viewing paradigm in the current study. On the other hand it must be noted that a contradictory mechanism is also possible: Since positive words sometimes have a social connotation, it cannot be ruled out that they induced anxiety in patients to a certain extent although we tried to minimize social connotations in all word categories apart from the SP-related one. This mechanism would also account for the higher anxiety induction ratings for positive words in the SP group. However, future research is necessary as results on positive-stimuli processing in social phobia seem generally mixed so far with several other studies that found no group differences for happy faces, or even used positive stimuli as a baseline for social-anxiety inducing paradigms (see [55]).

In accordance with the fMRI-results, the rating data showed group differences in valence, arousal and anxiety-induction ratings for SP-related but not for generally negative words. Interestingly, patients also rated neutral and positive words as more anxiety-inducing than HC, corroborating the view that anxiety patients tend to interpret many stimuli as more threatening than healthy persons [56]. As depicted in Table 3, higher negativity, arousal, and anxiety-induction ratings of SP-related but not generally negative words were strongly associated with higher symptom severity scores in the SPS and SIAS. While there were some positive associations between higher BDI, STAI-T, or STAI-S scores and negatively biased ratings for several word categories, social phobia severity seems to selectively influence ratings of SP-related words only. With the exception of arousal ratings for neutral words, this effect underlines the specificity of disorder-related information processing biases.

The functional connectivity results reveal a weaker connectivity in SP than in HC between the right amygdala and the left Brodmann Area 8 as part of the middle frontal gyrus. This area includes the frontal eye field (FEF), which is discussed to be related to orientation towards visual stimuli, as part of the attentional network (see e.g. [57]). Interestingly, this area has been reported to be deactivated in SP when anticipating social speaking [32], a result similar to ours.

Furthermore, more severely affected patients showed a weaker functional connectivity between the left amygdala and the ventromedial prefrontal (BA 10) as well as the orbitofrontal cortex (OFC, BA 11). The latter result is in accordance with Hahn and colleagues, who reported a negative association between state anxiety and resting-state connectivity between the amygdalae and the OFC in a sample of SP patients and HC [34]. The OFC is strongly connected to subcortical regions, and is assumed to play a crucial role in emotion regulation circuits [58], [59]. Thus, increased activation in the OFC or stronger connectivity between the amygdala and the OFC have been related to successfully decreasing the emotional impact of aversive pictures [60], [61]. In SP, decreased OFC activation was found in a social-speaking task [62], and there is evidence suggesting abnormalities in white matter tracts connecting the amygdala and the OFC [63]–[65]. The ventromedial cortex has also been related to emotion regulation [66]. Following [6], the BA 10 might be a higher-order executive brain region maintaining the goal to down regulate emotions, which is possibly carried out via OFC-amygdala connections.

Together, by using a paradigm consisting of emotional and disorder-specific words for the first time in SP, our results add a new piece of information to the existing knowledge on corticolimbic interaction patterns in SP. The disorder-dependent negative associations between the amygdala and both BA 10 and BA 11 support the view of an inverse relation between the prefrontal cortex and the amygdala that is disturbed in pathological anxiety (see [35], [59] for comparison). This finding has several clinical and methodological implications. First, a failure in prefrontal regulation, possibly resulting in amygdala hypersensitivity, might be a plausible neural correlate of the concentration on internal and external signs of threat, as postulated in models of SP (e.g. [67]). Second, there is evidence that altered prefrontal cortex activation in anxiety disorders can normalize after cognitive behavioral therapy [68], allowing us to understand better the action mechanism of psychotherapy. Finally, there are promising new therapeutic tools for anxiety disorders such as the repetitive transcranial magnetic stimulation (rTMS). As a future perspective, the impaired balance between cortical and subcortical areas might also be normalized via technically enhancing prefrontal activity [69]–[71]. From a methodological point of view, our data show the appropriateness of using verbal stimuli in fMRI research. As these stimuli are far better for distinguishing between general emotional or disorder-specific content than facial images, they provide new opportunities to study corticolimbic interactions in more detail, for example via psychophysiological interaction analysis or effective connectivity approaches.

We should point out some limitations of the current study. Although we included 25 persons per group, an even larger sample would have allowed uncovering subgroups within patients and controls, for example with respect to genetic factors. As in healthy subjects [72] and other psychiatric disorders [73], [74], for review see [75], there is also evidence for genetic influences on amygdala activation in social phobia [76], [77]. Furthermore, only female participants were included in the current sample. Although there are some studies reporting a balanced lifetime-prevalence for social phobia [78], the disorder is generally suggested to be more prevalent in women [40]. Thus including only female participants naturally limits the generalizability of our results, but it also reflects the disorder's gender distribution and avoids additional variance in our data due to differences in brain activations between men and women [41], [79]. Moreover, there are findings indicating that brain activation in crucial emotion-processing brain regions varies as a function of menstrual cycle [80], a variable which we did not account for. Finally, some participants were left-handed, which may constitute a problem with respect to the neural processing of verbal stimuli. Although the lateralization of limbic or frontal activation was of no interest for the goals of this investigation, and even though the number of left-handed participants was matched between the groups, this must be considered a limitation of the current study.

In sum, the current study emphasizes the important role of the limbic system and the prefrontal cortex as well as their interplay in social phobia. We used words as stimulus material to explicitly evaluate effects of disorder-specificity, over and above effects of general negativity of stimuli. With a functional connectivity analysis, we corroborated the view of an impaired functional coupling between the amygdala and prefrontal brain regions. Our results provide first evidence of a direct association between symptom severity and a weaker functional coupling between the amygdala and the ventromedial and orbitofrontal cortex during the confrontation with verbal emotional stimuli.

## Supporting Information

Table S1 Significant whole-brain results for the group x word category ANCOVA comparing activation to negative, SP-related, and positive (> neutral) words between patients with social phobia (SP) and healthy controls (HC).
(DOCX)
