# Supplementary material for: Of ‘Disgrace’ and ‘Pain’ – Corticolimbic Interaction Patterns for Disorder-Relevant and Emotional Words in Social Phobia
Source: PLoS One. 2014 Nov 14;9(11):e109949. doi: 10.1371/journal.pone.0109949 (PMC4232246; doi:10.1371/journal.pone.0109949)
Supplement: Table S1 — Significant whole-brain results for the group x word category ANCOVA comparing activation to negative, SP-related, and positive (> neutral) words between patients with social phobia (SP) and healthy controls (HC). (DOCX) [file pone.0109949.s001.docx]

**Table S1.** Significant whole-brain results for the group x word category ANCOVA comparing activation to negative, SP-related, and positive (> neutral) words between patients with social phobia (SP) and healthy controls (HC). The analysis was conducted at *p* = 0.001 uncorrected (corrected at p < 0.05 on the cluster level using the AlphaSim procedure, which resulted in an empirically determined cluster-extent threshold of *k* = 37 voxels for the whole brain).

| Anatomical region  [direction of post-hoc test] | Side | BA | Cluster Size | x | y | z | *F*_2_,_143_ | *p*-value |
| --- | --- | --- | --- | --- | --- | --- | --- | --- |
| *Main effect of word category* |  |  |  |  |  |  |  |  |
| MOG; IOG; CCG; LGG; FFG [SP-rel. > neg. / pos.] | L | 17-19 | 465 | - 18 | - 98 | - 6 | 31.63 | < 0.001 |
| CCG; LGG; Cerebellum [SP-rel. > neg. / pos.] | L | 18, 19, 29, 30, 31 | 226 | - 8 | - 68 | 10 | 12.83 | < 0.001 |
| Precuneus; Cuneus; CCG [SP-rel. / pos. > neg.] | L/R | 7, 23, 31 | 224 | 0 | - 64 | 34 | 12.01 | < 0.001 |
| CCG; LGG; PHG [SP-rel. > pos.] | R | 18, 19, 29, 30 | 121 | 22 | - 58 | 8 | 11.58 | < 0.001 |
| Medial frontal gyrus (orbital part); Rectal gyrus; ACC; olfactory gyrus [SP-rel. > neg.] | L/R | 10, 11, 24, 25, 32 | 166 | - 4 | 34 | - 6 | 11.42 | < 0.001 |
| FFG; PHG [SP-rel. > pos.] | L | 36, 37 | 37 | - 22 | - 38 | - 16 | 10.63 | 0.043 |
| STP; Insula [SP-rel. > pos.] | R | 21, 38 | 37 | 48 | 10 | - 18 | 9.72 | 0.043 |
| *Interaction effect group x word category* | |  |  |  |  |  |  |  |
| IOG; FFG; LGG [HC: neg. > SP-rel.] | R | 18 | 72 | 30 | - 84 | - 4 | 16.56 | < 0.001 |
| Cerebellum; FFG; ITG [HC: neg. > SP. rel.; SP > HC SP-rel.] | L | 18, 19, 37 | 323 | - 34 | - 64 | - 24 | 13.77 | < 0.001 |
| PHG; Hippocampus [HC: neg. > SP-rel.] | L | 28, 35 | 73 | - 18 | - 22 | - 14 | 13.1 | < 0.001 |
| Cerebellum [HC: neg. > SP-rel.] | R |  | 41 | 24 | - 58 | - 48 | 12.8 | 0.030 |
| STG [SP: SP-rel. > neg.] | R | 21, 22 | 49 | 56 | - 20 | 0 | 12.73 | 0.006 |
| Cerebellum [SP: SP-rel. > neg.] | R |  | 40 | 40 | - 78 | - 28 | 12.72 | 0.031 |
| MTG; STG [SP: SP-rel. > neg.] | L | 21, 22 | 46 | - 66 | - 28 | 2 | 11.69 | 0.012 |
| SPG; Precuneus; IPG [HC: neg. > SP-rel.] | L | 7 | 85 | - 18 | - 72 | 60 | 11.33 | < 0.001 |
| STG; Supramarginal gyrus [SP: SP-rel. > neg.] | L | 13, 40 | 40 | - 54 | - 42 | 20 | 10.08 | 0.031 |
| Cerebellum [HC: neg. > SP-rel.] | R |  | 63 | 36 | - 48 | - 28 | 9.96 | 0.001 |
| MTG; STG [SP: SP-rel. > neg.] | R | 22, 39 | 43 | 56 | - 52 | 10 | 9.91 | 0.020 |
| MTG; STG [HC: neg. > SP-rel.] | R | 21, 22 | 58 | 66 | - 46 | - 4 | 8.67 | 0.001 |

Coordinates are given in MNI space. MOG, middle occipital gyrus; IOG, inferior occipital gyrus; CCG, calcarine gyrus; LGG, lingual gyrus; FFG, fusiform gyrus; PHG, parahippocampal gyrus; ACC, anterior cingulate cortex; STP, superior temporal pole; ITG, inferior temporal gyrus; STG, superior temporal gyrus; MTG, middle temporal gyrus; SPG, superior parietal gyrus; IPG, inferior parietal gyrus.
